# Cancer stem cells as ‘units of selection’

**DOI:** 10.1111/eva.12017

**Published:** 2012-10-23

**Authors:** Mel Greaves

**Affiliations:** Division of Molecular Pathology, Haemato-Oncology Research Unit, The Institute of Cancer ResearchSutton, UK

**Keywords:** disease Biology, evolutionary Medicine, natural selection and contemporary evolution

## Abstract

Cancer development is widely recognized to be a somatic cell evolutionary process with complex dynamics and highly variable time frames. Variant cells and descendent subclones gain competitive advantage via their fitness in relation to micro-environmental selective pressures. In this context, the ‘unit’ of selection is the cell, but not any cell. The so-called ‘cancer stem cells’ have the essential properties required to function as the key units of selection, particularly with respect to their proliferative potential and longevity. These cells drive evolutionary progression of disease and provide reservoirs for relapse or recurrence and drug resistance. They represent the prime, but elusive and moving, targets for therapeutic control.

Cancers originate in single cells whose clonal progeny undergo successive rounds of genetic diversification and selection as they proliferate within tissue ecosystems (Nowell 1976; Gatenby and Vincent 2003; Merlo et al. 2006; Greaves and Maley 2012). The process is relatively inefficient; most tumours regress or remain indolent and clinically silent. Those that progress to overt malignancy do so over variable time frames spanning approximately 1 to 50 years. The culmination of this process, if not curtailed by successful treatment, is the emergence of a robust or weed-like quasispecies of cell that migrates, colonizes and hijacks other tissue territories with resultant demise of the host.

This behavioural trait in cells is empowered by acquired mutational and possibly epigenetic alterations in the genome that alter cellular phenotypes. Three per cent or so of genes in the human genome may, in total, contribute, as mutants, to the pathogenesis of cancer, the number of acquired mutations per cancer case varying between tens to thousands (Stratton et al. 2009). Of these, it is generally believed that only a modest number (perhaps approximately 5–10) contribute critically, or functionally, as ‘drivers’ of oncogenesis (Stratton et al. 2009); others, the great majority in most cases, are neutral or ‘passenger’ mutations whose allelic burden in the cancer cell populations reflects genetic instability, drift or hitchhiking on ‘drivers’. It has now become clear that the total genomic landscape of a cancer, as uncovered by sequencing, has an underlying pattern of segregation of mutations in which subclones have variegated mutational profiles; the only universally shared or common mutation in all subclones of any individual patient may then be the founder or ‘initiating’ mutation (Anderson et al. 2011; Navin et al. 2011; Gerlinger et al. 2012; Nik-Zainal et al. 2012). Moreover, genetically distinct subclones may occupy distinctive regions of the primary site (Clark et al. 2008; Gerlinger et al. 2012). This pattern of mutational complexity underscores the likelihood that cancers evolve not in a simple linear fashion but rather with a complex and branching clonal architecture, reminiscent of Darwin's iconic 1837 drawing of evolutionary speciation (Greaves and Maley 2012). The clinical implications of these patterns of genetic, subclonal diversity are substantial, particularly for biopsy-based prognosis and targeted therapeutics (Nowell 1976; Gerlinger et al. 2012; Greaves and Maley 2012).

The ‘driver’ mutations are considered to have an altered function and impact on cancer cell behaviour and to be adaptive, altering the fitness of cells in relation to the selective pressure to which they are exposed. ‘Driver’ status and contribution to fitness can however be ambiguous and context-dependent, with for example, epistatic interaction with other mutations or exposure to genotoxic insult (see below). Evidence for ‘driver’ mutations emerging via selection comes in the form of their recurrence in a series of cancers, a biased rate of nonsynonymous base pair changes [i.e. increased over that expected by chance alone (Youn and Simon 2011; Podlaha et al. 2012)] or by structural features (Bignell et al. 2010). Most cancer cells have multiple recurrent mutations with ‘driver’ credentials impacting on distinctive signalling pathways in cells, and the supposition is that the composite mutant genotype provides for particular adaptive phenotypes that directly or indirectly result in enhanced survival and/or proliferative activity (Hanahan and Weinberg 2011).

Selective pressures on cancer clone evolution operate within specialized, complex and dynamic tissue ecosystems (Gatenby and Gillies 2008; Pienta et al. 2008). Negative selective pressures will stall or slowdown tumour growth, at least initially, but also beget altered competitiveness of particular subclones or, under very stringent selective conditions, a selective sweep of one genetically distinct subclone. Several categories of selective pressures can be envisioned (Fig. 1). These pressures result in the emergence of adaptive traits or phenotypic changes in cancer cells that represent the so-called ‘hallmarks of cancer’ (Hanahan and Weinberg 2011). Metastasis represents the culmination of the evolutionary process, and as a single cell or clonal process (Yachida et al. 2010; Wu et al. 2012), it most likely involves both an evolutionary bottleneck in successful cell emigration and colonization plus the acquisition of adaptive traits that facilitate survival and proliferation in ectopic sites. In some instances, the match of mutation, adaptive trait and selective pressure are transparent, as for example with resistance to specific drugs (Shah et al. 2002; Balak et al. 2006) or with immune-editing and immune pressure (Dunn et al. 2002; Vago et al. 2009). The probability of a mutant trait existing will be constrained by the variables of mutation rate and clone size or cell number and perhaps tissue architecture (Nowak et al. 2003). The assumption is then that specific mutations arise randomly, predating exposure to the selective pressure for which they are, by chance, adaptive. In this context, neutral mutations can acquire context-specific ‘driver’ currency. Evidence for this is very limited but found for drug resistance (Roche-Lestienne et al. 2003; Diaz et al. 2012).

**Figure 1:**
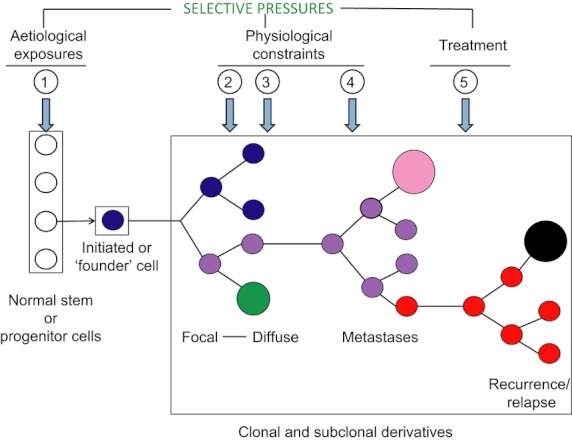
Selective pressures in cancer clone evolution. Coloured circles in box: genetically distinctive subclones of arbitrarily different sizes (i.e. variable sub-clonal dominance). (1–5) selective pressures. AT: adaptive traits. (1) Toxic or genotoxic cell damage. AT: selection for cells with adaptive mutations that enable genetic instability and/or a bypass cell cycle arrest, DNA repair or apoptosis (Bardelli et al. 2001). (2) Competition between different cancer cell clones or between cancer cells and normal cells for space and nutrient resources AT: loss of cell contact inhibition, paracrine or autocrine stimulation, rapid growth, inhibition of competitors. (3) Multiple physiological constraints, for example, default apoptosis signalling for cells with overt proliferative drive, anoxia, immune recognition. AT: bypass of apoptotic signals, solicitation of angiogenesis, immune-editing. (4) Multiple constraints on successful cell emigration from primary site, survival in lymphatics or blood, infiltration of ecotopic tissue and proliferation in that site. AT: Acquisition of migratory phenotype, adhesive/shape changes and adaptation to, or solicitation of, growth signals in ectopic sites. (5) Cell kill with treatment. AT: quiescence (- generic drug resistance); specific resistance via mutation in targets or pathways for drugs; bypass of drug target signalling requirements.

## What is the unit or hierarchical level of selection?

As the cancer clone evolves and negotiates a succession of selective pressures of variable stringency, the issue arises as to what exactly is being selected or at what hierarchical level selection is operating? This question is equivalent to the long-standing and contentious debate on units of selection in ecological evolution or speciation where philosophy, semantics and biological principles have enjoyed an uneasy relationship (Sober and Wilson 1994; Ridley 2003; Okasha 2006). Here, notions of group selection have effectively been surrendered to the more compelling case for individual organism selection. But this in turn has been challenged by George Williams (Williams 1992) and Richard Dawkins (1984) in particular who championed the gene or discrete genetic entities as the ultimate units of selection.

I adopt here what is perhaps the simplest or least contentious argument as advanced originally by Lewontin (1970). That is that the unit of evolutionary selection has the essential features of (i) phenotypic variation, (ii) differential fitness co-variant with a phenotypic trait and (iii) heritability; fitness being defined as survival and reproduction. Proliferation is self-evidently necessary for heritability, but in the context of cancer, the degree of replicative potential may be critical in trying to denote the effective units of selection.

Williams and Dawkins’ contention that single genetic genes or genetic loci are the units of selection is based on the argument that variant genes are the ultimate survivors (or replicators) as the genome as a whole is split by recombination, and the host individual (or ‘vehicle’ for replication) dies. This idea might appear to have currency for ‘selfish’ mutations in cancer, but ‘genes eye view’ has little force when considering cancer clone evolution. First, the latter is more akin to reproduction of an asexual, unicellular species. And, second, the adaptive traits in cancer are contingent upon mutant gene networks that critically involve deletions or loss of genetic information (and a loss of the restraints they encode). There is perhaps a philosophical argument that could be made for the shifting or evolving cancer cell genome (Yates and Campbell 2012) as the ultimate unit of selection as it out-survives individual cells, but more practically, the cell is the unit of selection and its clonal progeny the beneficiaries. It has been argued that somatic cells cannot be effective units of selection for a Weismannist species such as *H. sapiens* (i.e. where germ line and somatic cells are distinct) because any short-term advantage is lost when the host individual dies (Sober and Wilson 1994). This is unnecessarily restrictive when applied to cancer. The cancer clones evolve by a classical Darwinian process of natural selection is not negated by the stark fact that their host's demise also signals their end, any more than it would for short-term evolution of virulent human parasites and viruses (Levin and Bull 1994). In George Williams’ apt phrase, ‘evolution has no eyes to the future’ (Williams 1966). The short-term advantage of cancer cells can however, at least occasionally, be dramatically extended. A clone of cancer cells can enjoy variable degrees of selective advantage for decades but more strikingly can, under appropriate, albeit rare, circumstances, transit person to person (Greaves 2000; Isoda et al. 2009), persist in culture, as cell lines, for decades after the host's demise as exemplified by HeLa cells (Skloot 2010) or, in exceptional circumstances, persist and expand locally or globally over hundreds of years as a clonal, unicellular parasite (Murgia et al. 2006; Murchison 2009).

The major evolutionary transition to multi-cellularity involved the suppression of individual cells as units of selection within a more complex hierarchical organization in which the whole organism becomes the predominant unit of selection (Michod 1999). However, the capacity for clonal or cellular selection on a short-term or highly regulated basis is a preserved feature of more complex organisms. Embryogenesis, resilience of tissues, regenerative capacity, wound healing, specific immune responses and longevity all depend upon selective cell replication. Furthermore, some of the critical cells in these processes express telomerase that facilitates very extensive proliferative activity if not replicative immortality (Blasco 2005). There is, therefore, an inherent potential for natural selection at the level of somatic cells (Cairns 1975; Greaves 2000). Clearly, there are multiple evolved constraints that normally prohibit clonal escape; multi-cellularity would not have survived as a highly successful, emergent condition otherwise. But in this context, cancer reflects a loss of such controls, allowing a reversion to unicellular selfishness in which cells are the primary units of selection.

## But which cancer cells?

Cells as the units of evolutionary selection then, but does this mean any or all cancer cells expressing relevant phenotypic traits that are adaptive to negative selective pressure? The answer must be no, because of the heritability criteria for units of selection. Cancer cells that are genetically identical, that is, all members of the same subclone or clade, vary epigenetically in their replicative potential. Generally speaking, as progeny cells differentiate, they restrict their proliferative lifespan and then senesce or die. There is likely to be selective pressure in cancer development for cells which can undergo self-renewing proliferative cycles with no, or minimal, differentiation.

**Figure 2:**
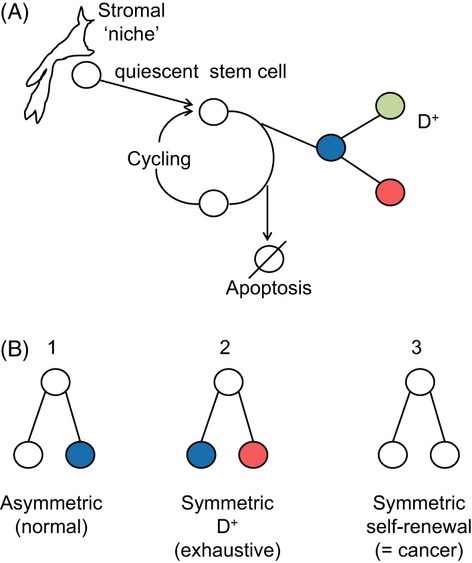
Functional state options for normal stem cells. Developmental options for normal stem cells. D^+^: differentiation of progeny cells. (A) Three potential outputs of cycling stem cells. Different coloured circles represent distinct differentiation or lineage pathways.

**Figure 3:**
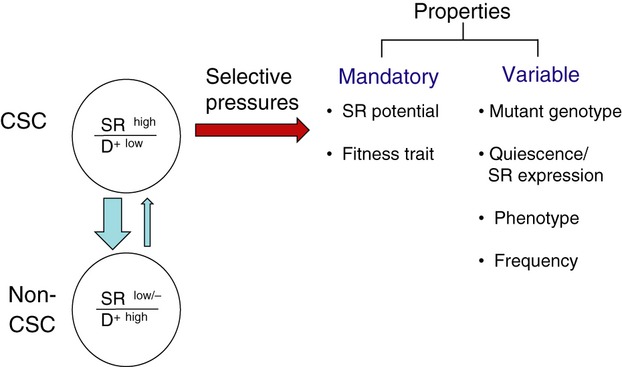
Properties of cancer stem cells. Although most selective pressures impose restraint on cancer cell proliferation or disease progression, occasionally these can also be positive. For example toxic exposures may result in a regenerative microenvironment, chronic inflammation can provide stimulus for clonal progression (Grivennikov et al. 2010) and genotoxic stimuli (including therapy) can increase mutational complexity and thereby the substrate for selection. Selective pressures can include environmentally derived genotoxicity, natural or physiological restraints, cancer therapy, and so on (Fig. 1). Mutation in progenitor cells or ecological pressures can convert these cells back to a self-renewing population (= small blue arrow); the large blue arrow represents differentiation: in both cases they represent a change in state. In addition to the mandatory trait of self-renewal, cancer stem cells (CSC) must exhibit a phenotypic trait that allows them to continue to survive and proliferate in the face of particular constraints or selective pressures. D^+^, differentiation; SR, self-renewal.

Cancer cells that self-renew are commonly referred to cancer stem cells (CSC), by analogy with normal stem cells that, by definition, also self-renew but under tightly regulated, ‘demand-led’, circumstances (Dick 2008). Normal stem cells can adopt several different states (Fig. 2). In cancer, cells with stem cell-like features are similarly adaptive but with a bias towards symmetrical (self-renewing) proliferative cycles, coupled with prohibition of differentiation and cell death (Cicalese et al. 2009). Cells with these features probably evolve from rare to very common (within a clone) as the disease progresses, although quantitative evidence for this is still limited. Certainly, the frequency of cancer stem cells, as assayed by transplantation in immune-deficient mice, varies from very low (approximately 1 in 10^6^) (Ishizawa et al. 2010; Sarry et al. 2011) to very high (approximately 1 in 4) (Quintana et al. 2008). This may reflect, in part, different cancers with distinctive genetic abnormalities but also stage of disease (Driessens et al. 2012). The human cancer stem cell field has been highly contentious, in part because of uncertainties over the efficiency and applicability of the *in vivo* immune-deficient mouse xeno-transplantation assays used but also because of variable data on CSC frequency, immunophenotype, proliferative rates and drug sensitivity [reviewed in (Rosen and Jordan 2009; Shackleton et al. 2009; Clevers 2011)]. The credibility of the CSC concept has however been boosted by the recent demonstration, using *in vivo* models of murine cancer, that infrequent cells with CSC-like properties can be tracked and are responsible for post-therapy recurrence (Chen et al. 2012; Driessens et al. 2012). The numerical and phenotypic discrepancies in CSC can effectively be accommodated by a relatively simple or minimal definition of a CSC, which only requires this cell to have the potential for extensive self-renewal cycles (O'Brien et al. 2010; Fig. 3). All other properties and frequencies of these cells can be expected to vary substantially, both contemporaneously between subclones and over time as the disease progresses. This applies to the mutant genetic profiles of CSC that are variable between subclones within individual patients with leukaemia (Anderson et al. 2011; Notta et al. 2011). The properties of extensive heritability of genotype/adaptive traits coupled with genotypic variability (in ‘driver’ mutations) provide a potent argument for CSC being the principal units of selection in cancer progression (Anderson et al. 2011; Greaves 2011). This argument does not exclude the fact that CSC'ness is a state, not a fixed entity (Gupta et al. 2011), that CSC often demonstrates ‘niche’ dependence (Beck et al. 2011; Malanchi et al. 2012) and that cells that would normally be considered differentiating progenitors can, under particular ecological conditions, for example, hypoxia (Koh et al. 2011), epithelial-mesenchymal transition (Gupta et al. 2009; Chaffer et al. 2011) or when particular mutations accrue, switch on or acquire a CSC potential. This altered fate is something that normal progenitors can do under regenerative stress, so it is unsurprising that cancer cells can be similarly flexible.

An adaptive trait, such as specific drug resistance, can arise randomly in any cancer cell – stem cell, progenitor or differentiate progeny. Indeed, it is statistically more likely to arise in nonstem cells because of their numerical superiority. And, any such cell would have a survival benefit in the context of the relevant selective pressure. But this is where the heritability argument for units of selection applies. Any benefit would be temporary or very transient for progenitors and differentiating cells and only sustained in the clonal progeny of cells with extensive self-renewal potential, that is, cancer stem cells. The protracted natural history of invasive, cancerous clones can be portrayed as essentially driven by repetitive diversification and selection of stem cells (Greaves 2010).

What is the normal cell that is initially selected by aetiological events or exposures as the founder for the cancer clone and its downstream, multiple subclones? The weight of evidence indicates that this cell is either a normal stem cell or a progenitor that acquires a stem cell state as a consequence of the founder genetic lesion *and/or* local ecological stress (Cozzio et al. 2003; Visvader 2011).

The notion that a limited (but highly variable) number of stem-like cells are the primary units of selection in cancer accords with the biological behaviour of most cancers and carries substantial clinical implications. The genetic (and possibly epigenetic) diversity of cancer stem cells is critical. Malignant cancers may manifest with single dominant clones, but there is always underlying clonal diversity (Nik-Zainal et al. 2012) sustained, we assume, by genetically distinct stem cells. As selective pressures change, so subclones with winning traits gain advantage. And these are not necessarily cells from within previously dominant subclones. Thus, both clonal metastasis (Yachida et al. 2010; Wu et al. 2012) and disease recurrence or relapse (Mullighan et al. 2008; Anderson et al. 2011; Clappier et al. 2011) can be backtracked to an earlier origin from minor subclones. In this context, the persistence of genetically variant cancer stem cells provides a critical reservoir for progression of disease and escape from therapy via clonal selection. It follows that the more genetically variable (and perhaps epigenetically) these cells are within a patient, the more likelihood there should be of malignant progression. The more numerous these cells are, the higher probability there should be of treatment failure via the selection of pre-existing resistant subclones. Direct evidence to support these contentions is limited, but there are reported associations between poor treatment outcome in cancer and various measures of ‘stemness’, for example, self-renewal signatures, rapidity of regeneration in transplants (van Rhenen et al. 2005; Eppert et al. 2011; Merlos-Suárez et al. 2011). Paradoxically, although CSC have the capacity for extensive or unlimited self-renewing proliferative cycles, they, in common with normal stem cells, can adopt a quiescent, out-of-cycle state, perhaps in association with particular stromal niches (Lane et al. 2009). This renders them significantly less vulnerable to chemo- or radio-therapy (Graham et al. 2002). These observations endorse the view that CSCs, as the likely units of selection, are also the critical cellular units for therapeutic attack or control. Unfortunately, they provide an elusive and moving target which may, at least in part, explain the intransigence of advanced disease.

## References

[b1] Anderson K, Lutz C, van Delft FW, Bateman CM, Guo Y, Colman SM, Kempski H (2011). Genetic variegation of clonal architecture and propagating cells in leukaemia. Nature.

[b2] Balak MN, Gong Y, Riely GJ, Somwar R, Li AR, Zakowski MF, Chiang A (2006). Novel D761Y and common secondary T790M mutations in epidermal growth factor receptor-mutant lung adenocarcinomas with acquired resistance to kinase inhibitors. Clinical Cancer Research.

[b3] Bardelli A, Cahill DP, Lederer G, Speicher MR, Kinzler KW, Vogelstein B, Lengauer C (2001). Carcinogen-specific induction of genetic instability. Proceedings of the National Academy of Sciences of the United States of America.

[b4] Beck B, Driessens G, Goossens S, Youssef KK, Kuchnio A, Caauwe A, Sotiropoulou PA (2011). A vascular niche and a VEGF-Nrp1 loop regulate the initiation and stemness of skin tumours. Nature.

[b5] Bignell GR, Greenman CD, Davies H, Butler AP, Edkins S, Andrews JM, Buck G (2010). Signatures of mutation and selection in the cancer genome. Nature.

[b6] Blasco MA (2005). Telomeres and human disease: ageing, cancer and beyond. Nature Reviews Genetics.

[b7] Cairns J (1975). Mutation selection and the natural history of cancer. Nature.

[b8] Chaffer CL, Brueckmann I, Scheel C, Kaestli AJ, Wiggins PA, Rodrigues LO, Brooks M (2011). Normal and neoplastic nonstem cells can spontaneously convert to a stem-like state. Proceedings of the National Academy of Sciences of the United States of America.

[b9] Chen J, Li Y, Yu T-S, McKay RM, Burns DK, Kernie SG, Parada LF (2012). A restricted cell population propagates glioblastoma growth after chemotherapy. Nature.

[b10] Cicalese A, Bonizzi G, Pasi CE, Faretta M, Ronzoni S, Giulini B, Brisken C (2009). The tumor suppressor *p53* regulates polarity of self-renewing divisions in mammary stem cells. Cell.

[b11] Clappier E, Gerby B, Sigaux F, Delord M, Touzri F, Hernandez L, Ballerini P (2011). Clonal selection in xenografted human T cell acute lymphoblastic leukemia recapitulates gain of malignancy at relapse. Journal of Experimental Medicine.

[b12] Clark J, Attard G, Jhavar S, Flohr P, Reid A, De-Bono J, Eeles R (2008). Complex patterns of *ETS* gene alteration arise during cancer development in the human prostate. Oncogene.

[b13] Clevers H (2011). The cancer stem cell: premises, promises and challenges. Nature Medicine.

[b14] Cozzio A, Passegué E, Ayton PM, Karsunky H, Cleary ML, Weissman IL (2003). Similar MLL-associated leukemias arising from self-renewing stem cells and short-lived myeloid progenitors. Genes and Development.

[b15] Dawkins R, Brandon RN, Burian R (1984). Replicators and vehicles. Genes, Organisms, Populations: Controversies Over the Units of Selection.

[b16] Diaz LA, Williams RT, Wu J, Kinde I, Hecht JR, Berlin J, Allen B (2012). The molecular evolution of acquired resistance to targeted EGFR blockade in colorectal cancers. Nature.

[b17] Dick JE (2008). Stem cell concepts renew cancer research. Blood.

[b18] Driessens G, Beck B, Caauwe A, Simons BD, Blanpain C (2012). Defining the mode of tumour growth by clonal analysis. Nature.

[b19] Dunn GP, Bruce AT, Ikeda H, Old LJ, Schreiber RD (2002). Cancer immunoediting: from immuno-surveillance to tumor escape. Nature Immunology.

[b20] Eppert K, Takenaka K, Lechman ER, Waldron L, Nilsson B, Metzeler P, van Galen KH (2011). Stem cell gene expression programs influence clinical outcome in human leukemia. Nature Medicine.

[b21] Gatenby RA, Gillies RJ (2008). A microenvironmental model of carcinogenesis. Nature Reviews Cancer.

[b22] Gatenby RA, Vincent TL (2003). An evolutionary model of carcinogenesis. Cancer Research.

[b23] Gerlinger M, Rowan AJ, Horswell S, Larkin J, Endesfelder D, Gronroos E, Martinez P (2012). Intratumor heterogeneity and branched evolution revealed by multiregion sequencing. New England Journal of Medicine.

[b24] Graham SM, Jørgensen HG, Allan E, Pearson C, Alcorn MJ, Richmond L, Holyoake TL (2002). Primitive, quiescent, Philadelphia-positive stem cells from patients with chronic myeloid leukemia are insensitive to ST1571 *in vitro*. Blood.

[b25] Greaves M (2000). Cancer. The Evolutionary Legacy.

[b26] Greaves M (2010). Cancer stem cells: back to Darwin?. Seminars in Cancer Biology.

[b27] Greaves M (2011). Cancer stem cells renew their impact. Nature Medicine.

[b28] Greaves M, Maley CC (2012). Clonal evolution in cancer. Nature.

[b29] Grivennikov SI, Greten FR, Karin M (2010). Immunity, inflammation, and cancer. Cell.

[b30] Gupta PB, Chaffer CL, Weinberg RA (2009). Cancer stem cells: mirage or reality?. Nature Medicine.

[b31] Gupta PB, Fillmore CM, Jiang G, Shapira SD, Tao K, Kuperwasser C, Lander ES (2011). Stochastic state transitions give rise to phenotypic equilibrium in populations of cancer cells. Cell.

[b32] Hanahan D, Weinberg RA (2011). Hallmarks of cancer: the next generation. Cell.

[b33] Ishizawa K, Rasheed ZA, Karisch R, Wang Q, Kowalski J, Susky E, Pereira K (2010). Tumor-initiating cells are rare in many human tumors. Cell Stem Cell.

[b34] Isoda T, Ford AM, Tomizawa D, Van Delft FW, Gonzalez De Castro D, Mitsuiki N, Score J (2009). Immunologically silent cancer clone transmission from mother to offspring. Proceedings of the National Academy of Sciences of the United States of America.

[b35] Koh MY, Lemos R, Liu X, Powis G (2011). The hypoxia-associated factor switches cells from HIF-1a- to HIF-2a-dependent signaling promoting stem cell characteristics, aggressive tumor growth and invasion. Cancer Research.

[b36] Lane SW, Scadden DT, Gilliland DG (2009). The leukemic stem cell niche - current concepts and therapeutic opportunities. Blood.

[b37] Levin BR, Bull JJ (1994). Short-sighted evolution and the virulence of pathogenic microorganisms. Trends in Microbiology.

[b38] Lewontin RC (1970). The units of selection. Annual Review of Ecology and Systematics.

[b39] Malanchi I, Santamaria-Martínez A, Susanto E, Peng H, Lehr H-A, Delaloye J-F, Huelsken J (2012). Interactions between cancer stem cells and their niche govern metastatic colonization. Nature.

[b40] Merlo LMF, Pepper JW, Reid BJ, Maley CC (2006). Cancer as an evolutionary and ecological process. Nature Reviews Cancer.

[b41] Merlos-Suárez A, Barriga FM, Jung P, Iglesias M, Virtudes Céspedes M, Rossell D, Sevillano M (2011). The intestinal stem cell signature identifies colorectal cancer stem cells and predicts disease relapse. Cell Stem Cell.

[b42] Michod RE (1999). Darwinian Dynamics: Evolutionary Transitions in Fitness and Individuality.

[b43] Mullighan CG, Phillips LA, Su X, Ma J, Miller CB, Shurtleff SA, Downing JR (2008). Genomic analysis of the clonal origins of relapsed acute lymphoblastic leukemia. Science.

[b44] Murchison EP (2009). Clonally transmissible cancers in dogs and Tasmanian devils. Oncogene.

[b45] Murgia C, Pritchard JK, Kim SY, Fassati A, Weiss RA (2006). Clonal origin and evolution of a transmissible cancer. Cell.

[b46] Navin N, Kendall J, Troge J, Andrews P, Rodgers L, McIndoo J, Cook K (2011). Tumour evolution inferred by single-cell sequencing. Nature.

[b47] Nik-Zainal S, Van Loo P, Wedge DC, Alexandrov LB, Greenman CD, Lau KW, Raine K, Breast Cancer Working Group of the International Cancer Genome Consortium Cell (2012). The life history of 21 breast cancers. Cell.

[b48] Notta F, Mullighan CG, Wang JC, Poeppl A, Doulatov S, Phillips LA, Ma J (2011). Evolution of human *BCR-ABL1* lymphoblastic leukaemia-initiating cells. Nature.

[b49] Nowak MA, Michor F, Iwasa Y (2003). The linear process of somatic evolution. Proceedings of the National Academy of Sciences of the United States of America.

[b50] Nowell PC (1976). The clonal evolution of tumor cell populations. Science.

[b51] O'Brien CA, Kreso A, Jamieson CHM (2010). Cancer stem cells and self-renewal. Clinical Cancer Research.

[b52] Okasha S (2006). Evolution and the Levels of Selection.

[b53] Pienta KJ, McGregor N, Axelrod R, Axelrod DE (2008). Ecological therapy for cancer: defining tumors using an ecosystem paradigm suggests new opportunities for novel cancer treatments. Translational Oncology.

[b54] Podlaha O, Riester M, De S, Michor F (2012). Evolution of the cancer genome. Trends in Genetics.

[b55] Quintana E, Shackleton M, Sabel MS, Fullen DR, Johnson TM, Morrison SJ (2008). Efficient tumour formation by single human melanoma cells. Nature.

[b56] van Rhenen A, Feller N, Kelder A, Westra AH, Rombouts E, Weegman SZ, van der Pol MA (2005). High stem cell frequency in acute myeloid leukemia at diagnosis predicts high minimal residual disease and poor survival. Clinical Cancer Research.

[b57] Ridley M (2003). Evolution.

[b58] Roche-Lestienne C, Laï J-L, Darré S, Facon T, Preudhomme C (2003). A mutation conferring resistance to imatinib at the time of diagnosis of chronic myelogenous leukemia. New England Journal of Medicine.

[b59] Rosen JM, Jordan CT (2009). The increasing complexity of the cancer stem cell paradigm. Science.

[b60] Sarry J-E, Murphy K, Perry R, Sanchez PV, Secreto A, Keefer C, Swider CR (2011). Human acute myelogenous leukemia stem cells are rare and heterogeneous when assayed in NOD/SCID/IL2Rgc-deficient mice. Journal of Clinical Investigation.

[b61] Shackleton M, Quintana E, Fearon ER, Morrison SJ (2009). Heterogeneity in cancer: cancer stem cells versus clonal evolution. Cell.

[b62] Shah NP, Nicoll JM, Nagar B, Gorre ME, Paquette RL, Kuriyan J, Sawyers CL (2002). Multiple BCR-ABL kinase domain mutations confer polyclonal resistance to the tyrosine kinase inhibitor imatinib (STI571) in chronic phase and blast crisis chronic myeloid leukemia. Cancer Cell.

[b63] Skloot R (2010). The Immortal Life of Henrietta Lacks.

[b64] Sober E, Wilson DS (1994). A critical review of philosophical work on the units of selection problem. Philosophy of Science.

[b65] Stratton MR, Campbell PJ, Futreal PA (2009). The cancer genome. Nature.

[b66] Vago L, Perna SK, Zanussi M, Mazzi B, Barlassina C, Stanghellini MTL, Perrelli NF (2009). Loss of mismatched HLA in leukemia after stem-cell transplantation. New England Journal of Medicine.

[b67] Visvader JE (2011). Cells of origin in cancer. Nature.

[b68] Williams GC (1966). Adaptation and Natural Selection.

[b69] Williams GC (1992). Natural Selection: Domains, Levels, Challenges.

[b70] Wu X, Northcott PA, Dubuc A, Dupuy AJ, Shih DJH, Witt H, Croul S (2012). Clonal selection drives genetic divergence of metastatic medulloblastoma. Nature.

[b71] Yachida S, Jones S, Bozic I, Antal T, Leary R, Fu B, Kamiyama M (2010). Distant metastasis occurs late during the genetic evolution of pancreatic cancer. Nature.

[b72] Yates LR, Campbell PJ (2012). Evolution of the cancer genome. Nature Reviews Genetics.

[b73] Youn A, Simon R (2011). Identifying cancer driver genes in tumor genome sequencing studies. Bioinformatics.

